# Mortality at older ages and moves in residential and sheltered housing: evidence from the UK

**DOI:** 10.1136/jech-2013-203097

**Published:** 2014-03-17

**Authors:** James Robards, Maria Evandrou, Jane Falkingham, Athina Vlachantoni

**Affiliations:** 1 EPSRC Care Life Cycle, Social Sciences, University of Southampton, Southampton, Hampshire, UK; 2 ESRC Centre for Population Change, Social Sciences, University of Southampton, Southampton, Hampshire, UK; 3 Centre for Research on Ageing, Social Sciences, University of Southampton, Southampton, Hampshire, UK

**Keywords:** AGEING, MORTALITY, MARITAL STATUS, HOUSING

## Abstract

**Background:**

The study examines the relationship between transitions to residential and sheltered housing and mortality. Past research has focused on housing moves over extended time periods and subsequent mortality. In this paper, annual housing transitions allow the identification of the patterning of housing moves, the duration of stay in each sector and the assessment of the relationship of preceding moves to a heightened risk of dying.

**Methods:**

The study uses longitudinal data constructed from pooled observations from the British Household Panel Survey (waves 1993–2008). Records were pooled for all cases where the survey member is 65 years or over and living in private housing at baseline and observed at three consecutive time points, including baseline (N=23 727). Binary logistic regression (death as outcome three waves after baseline) explored the relative strength of different housing transitions, controlling for sociodemographic predictors.

**Results:**

(1) Transition to residential housing within the previous 12 months was associated with the highest mortality risk. (2) Results support existing findings showing an interaction between marital status and mortality, whereby unmarried persons were more likely to die. (3) Higher male mortality was observed across all housing transitions.

**Conclusions:**

An older person's move to residential housing is associated with a higher risk of mortality within 12 months of the move. Survivors living in residential housing for more than a year, show a similar probability of dying to those living in sheltered housing. Results highlight that it is the type of accommodation that affects an older person's mortality risk, and the length of time they spend there.

## Introduction

Understanding the mortality risk associated with different housing ‘pathways’ among older people is crucial in planning for the future demand for long-term care housing and services.^1^ In the UK, housing transitions and subsequent mortality among older people have been studied over 10 year intervals with the use of census data.^2–6^ Research on mortality and housing transitions over shorter timescales at older ages has focused on palliative care.^7^ ^8^ Transitions into residential housing have been studied in relation to solo living,^9^ ^10^ demographic factors^6^ ^11^ ^12^ and socioeconomic characteristics.^4^ However, relatively little research has examined moves from the private sector into sheltered and residential housing and how such moves are associated with subsequent mortality risk. Residential housing is a type of living arrangement where older persons with physical and/or mental frailty move into a residential home providing board and personal care 24 h a day, 7 days a week following the assessment of their needs. Sheltered housing usually takes the form of a group of small bungalows or flats supervised by a Scheme Manager, who can offer help in an emergency. Estimating mortality risk is important for understanding the duration of time spent in different housing types and the mortality risk associated with any such move. Therefore, the present study details year-on-year transitions between housing sectors (private household, sheltered, residential) for persons 65 years and over in order to examine the relationship between such moves in the preceding 3 years and the heightened risk of mortality, taking into account demographic, health and socioeconomic characteristics.

Analyses using longitudinal data to consider long-term transitions and mortality have identified mortality outcomes associated with residential housing residence recorded at the previous census. Higher mortality among residents in long-term care institutions compared with those living alone, in a couple or with relatives has been found, with 26% of male and 36% of female institutional residents in 2001 surviving for 3 years.^2^ However, such research has tended to focus on residential care accommodation, and not included residents in sheltered accommodation.

The present study seeks to bridge the gap between the existing approaches to considering preceding housing transitions (long-term vs short-term change) and also take into account socioeconomic characteristics to estimate mortality risk among British Household Panel Survey (BHPS) members over the 1993–2008 time period. (Years 1991 and 1992 were excluded from the analysis as there is no information on transitions into sheltered housing at these waves). Existing work has emphasised the relationship between older persons’ health and their housing circumstances.^13^ ^14^ However, the relationship between housing transitions and mortality can often be mediated by changes in health status, caring arrangements^15^ and changes in marital status.^16^ Proactive residential relocation in later life considering or anticipating future events or stressors has been found to predominate among older people who are in younger age groups, more highly educated and from higher income groups.^17^ Residential and sheltered housing transitions for persons 65 years and older have been considered for the UK using BHPS data which offers detail on the household characteristics.^18^

## Aims

The aims of this study are (1) to identify the relationship between housing pathways and subsequent mortality; (2) to estimate the impact of duration in each housing type in relation to subsequent mortality; and (3) to disaggregate results by men and women. An overarching aim is to study mortality in relation to residential and sheltered housing transitions over a 3 year period rather than months (as in palliative care literature) or decades (as in some longitudinal analyses). The most important and insightful explanatory variable in our model combines moves out of the private sector into residential and sheltered housing, with the duration in that sector over a 3 year period.

## Data and methods

The study is based on pooled data from the BHPS (waves 1993–2008). Data are collected at individual and household levels and the survey asks a range of questions on demographic and socioeconomic characteristics, health status, housing type and other indicators.^19^ Attrition among BHPS members does not present substantial bias.^20^ Our analysis was restricted to persons 65 years and over, living in private housing at their first observation and who were observed at three consecutive time points (N=23 727). Our sample considers only those aged 65 years and over because below this age there are smaller numbers of older people transitioning to residential or sheltered housing. BHPS members aged 65 years and over were extracted from wave 1 and the cases were matched with responses to the same variables from waves 2 and 3. This process of identifying persons aged 65 years and over at the first of three successive waves continued until all waves were merged together (eg, waves 2, 3 and 4). The samples were stacked to create one large dataset of individuals and their responses. This data structure provides greater statistical power but there is potential for clustering of observations within cases and therefore for non-independence of observations^21^; robust SEs were calculated to allow for non-independence of observations.

**Figure 1:**
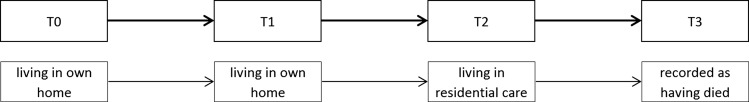
Diagram of longitudinal construction of British Household Panel Survey (BHPS) data, displaying an exemplar trajectory.

To show housing pathways between sectors, a cross wave housing trajectory variable was created from the housing variable for each wave. Figure 1 shows an example trajectory for a BHPS member who has one spell in residential accommodation before mortality between times T2 and T3 (end line measurement) while table 1 illustrates transitions into residential and sheltered housing and the way in which these are identified using the cross wave housing trajectory variable. Housing type was recorded at each wave, approximately 12 months apart. Therefore, because the date of movement into the type of housing is not precisely known, exact exposure to risk cannot be identified and instead a grouped time variable is used within which a transition occurred. The cross wave housing trajectory variable provides a more detailed set of housing transitions compared with those reported in previous studies.

**Table 1:** Cross wave housing trajectory variable

	Measurement		
Housing transition variable	T0	T1	T2
No transition—in private housing at all waves	Private	Private	Private
Transition to residential housing 2 years prior to end line measurement	Private	Residential	Residential
Transition to residential housing 1 year prior to end line measurement	Private	Private	Residential
Transition to sheltered housing 2 years prior to end line measurement	Private	Sheltered	Sheltered
Transition to sheltered housing 1 year prior to end line measurement	Private	Private	Sheltered
All others	Private	Sheltered and residential housing.	

The outcome measure (at T3) was the respondent's death three waves after the baseline. Mortality was identified from the variable on the latest known sample status of the individual and recorded in the dataset in a binary form. Binary logistic regression examined the relative impact on mortality of the duration of stay and the type of housing moved into, after controlling for other predictors of mortality. Our model building strategy was to include the housing transition variable and progressively include variables to control for demographic characteristics, health status and socioeconomic status. Analyses were completed in SPSS (V.19).

The model building strategy takes into account time (BHPS wave), demographic characteristics, housing transitions, health status and socioeconomic indices. All control variables are measured at baseline (T0) and are as reported by the BHPS member.

*BHPS wave*—controls for period effects.

*Cross wave housing trajectory variable*—timing of transition to sheltered or residential housing (table 1) (derived from housing type at each wave (T0, T1 and T2)).

### Demographic characteristics

*Age*—controls for differential mortality risk across different age groups.^22^

*Sex*—controls for differential mortality by sex.^23^

*Marital status—*controls for the accumulated risk of mortality at older ages in relation to marital dissolution and notions of marital protection,^24^ marital selection^25–27^ or potential spousal caring roles.^28^ The variable is the marital status at T0.

*Health status—*controls for the impact of self-reported health status/general state of health on mortality risk (the variable is derived from one's reported general health status in the last 12 months).

### Socioeconomic measures

*Employment category—*controls for a respondent's most recent job and is an indicator of social class. We considered income measure to be less suitable for the analysis as the sample encompasses persons who are above the state pension age. A socioeconomic class variable (most recent job) is used to classify employment into four standard groups for comparison.

*Household access to a car—*controls for the impact of housing wealth and mobility.

Coefficients from the final model are used to calculate predicted probabilities of mortality between T2 and T3 for men and women for a given housing transition type.^29^ The illustrated example combines the strongest predictors of mortality to illustrate the worst-case scenario (calculations were performed in STATA V.12 using ‘margins’).

Variables measuring health status at different time points were tested in the final model. The use of a measure of health status in the wave preceding any move (eg, T1 if in residential housing at T2) and in the wave after the move (eg, T2 if living in private housing at T1 and residential housing at T2) led to coefficients comparable with those resulting from the inclusion of one's health status at baseline (T0). Modest changes in housing transition coefficients were identified, however the overall direction of coefficients remained the same.

## Results

### Descriptive results

Column 1 in table 2 shows the distribution of the sample. Women comprised 57% (13 480) of the sample and a similar proportion of the sample were married or living as a couple, compared with widowed, separated or divorced persons (37%) and single never-married persons (7%). From the 23 727 persons aged 65 years and older living in private housing at baseline, 111 (0.5%) were living in residential housing after two waves while 202 (0.8%) moved to sheltered housing within the same time period.

**Table 2:** Odds ratios (and 95% CIs) for mortality at T3 by key covariates including housing transition

			Model 1			Model 2			Model 3			Model 4			Model 5			Model 6		
	N	%	Exp(B)	Sig.	0.95 CI	Exp(B)	Sig.	0.95 CI	Exp(B)	Sig.	0.95 CI	Exp(B)	Sig.	0.95 CI	Exp(B)	Sig.	0.95 CI	Exp(B)	Sig.	0.95 CI
Housing transition variable																				
No transition—in private housing at all waves	23 318	98.3	1.00			1.00			1.00			1.00			1.00			1.00		
Transition to residential housing 2 years prior to end line measurement	42	0.2	7.75	0.000	3.79 to 15.87	3.52	0.001	1.68 to 7.4	3.39	0.001	1.61 to 7.13	2.35	0.026	1.11 to 5	2.34	0.027	1.1 to 4.96	2.31	0.030	1.09 to 4.9
Transition to residential housing 1 year prior to end line measurement	69	0.3	9.45	0.000	5.53 to 16.15	4.68	0.000	2.67 to 8.21	4.59	0.000	2.62 to 8.04	3.83	0.000	2.18 to 6.71	3.75	0.000	2.14 to 6.59	3.72	0.000	2.12 to 6.53
Transition to sheltered housing 2 years prior to end line measurement	80	0.3	3.24	0.001	1.61 to 6.52	2.22	0.029	1.09 to 4.53	2.14	0.037	1.05 to 4.37	1.85	0.099	0.89 to 3.84	1.82	0.109	0.88 to 3.77	1.78	0.121	0.86 to 3.7
Transition to sheltered housing 1 year prior to end line measurement	122	0.5	1.55	0.264	0.72 to 3.33	1.09	0.826	0.5 to 2.38	1.06	0.876	0.49 to 2.32	0.99	0.987	0.45 to 2.19	1.00	1.000	0.46 to 2.2	0.98	0.950	0.44 to 2.14
All others	96	0.4	1.68	0.221	0.73 to 3.85	1.21	0.666	0.52 to 2.81	1.16	0.736	0.5 to 2.7	1.01	0.975	0.43 to 2.38	0.99	0.987	0.42 to 2.33	0.98	0.971	0.42 to 2.31
Age																				
65–74 years	14 410	60.7				1.00			1.00			1.00			1.00			1.00		
75–79 years	5042	21.3				1.97	0.000	1.65 to 2.34	1.92	0.000	1.61 to 2.29	1.88	0.000	1.58 to 2.25	1.90	0.000	1.6 to 2.27	1.87	0.000	1.57 to 2.24
80–84 years	2929	12.3				3.00	0.000	2.5 to 3.61	2.88	0.000	2.39 to 3.48	2.83	0.000	2.34 to 3.42	2.91	0.000	2.41 to 3.53	2.81	0.000	2.32 to 3.41
85–89 years	1071	4.5				5.54	0.000	4.45 to 6.9	5.18	0.000	4.12 to 6.5	5.11	0.000	4.06 to 6.42	5.23	0.000	4.16 to 6.59	4.97	0.000	3.93 to 6.27
90+ years	275	1.2				8.54	0.000	6.06 to 12.02	7.82	0.000	5.5 to 11.1	8.66	0.000	6.07 to 12.35	8.88	0.000	6.21 to 12.68	8.34	0.000	5.82 to 11.96
Sex																				
Male	10 247	43.2				1.00			1.00			1.00			1.00			1.00		
Female	13 480	56.8				0.68	0.000	0.59 to 0.77	0.64	0.000	0.55 to 0.73	0.59	0.000	0.51 to 0.69	0.62	0.000	0.53 to 0.71	0.59	0.000	0.51 to 0.69
Marital status																				
Married or living as a couple	13 301	56.1							1.00			1.00			1.00			1.00		
Widowed separated or divorced	8844	37.3							1.19	0.028	1.02 to 1.4	1.15	0.082	0.98 to 1.35	1.13	0.133	0.96 to 1.33	1.11	0.227	0.94 to 1.3
Never married	1582	6.7							1.44	0.006	1.11 to 1.86	1.42	0.008	1.1 to 1.84	1.41	0.009	1.09 to 1.82	1.38	0.014	1.07 to 1.79
Health status																				
Excellent	3265	13.8										1.00			1.00			1.00		
Good or very good (for general)	10 701	45.1										1.16	0.265	0.89 to 1.5	1.15	0.293	0.89 to 1.49	1.13	0.364	0.87 to 1.47
Fair	6925	29.2										1.89	0.000	1.46 to 2.45	1.85	0.000	1.43 to 2.4	1.78	0.000	1.37 to 2.31
Poor or very poor (for health status)	2836	12.0										3.89	0.000	2.97 to 5.09	3.81	0.000	2.91 to 5	3.62	0.000	2.75 to 4.76
Socioeconomic employment category																				
Non-manual	11 377	47.9													1.00			1.00		
Manual and unskilled	10 283	43.3													1.19	0.017	1.03 to 1.38	1.16	0.053	1 to 1.34
Missing and armed forces	899	3.8													1.00	0.984	0.71 to 1.41	0.71	0.154	0.45 to 1.14
Never had a job	1168	4.9													0.75	0.105	0.53 to 1.06	0.75	0.095	0.53 to 1.05
Access to a car																				
Yes	11 998	50.6																1.00		
No	6274	26.4																1.19	0.057	1 to 1.43
Do not drive	5075	21.4																1.20	0.070	0.99 to 1.46
Missing/wild/refused /not answered/proxy	380	1.6																2.10	0.009	1.21 to 3.65

Model 1: housing transition, wave. Model 2: housing transition, age, sex, wave. Model 3: age, sex, marital status, housing transition, wave. Model 4: age, sex, marital status, housing transition, health status, wave. Model 5: age, sex, marital status, housing transition, health status, socioeconomic employment category, wave. Model 6: age, sex, marital status, housing transition, health status, socioeconomic employment category, access to a car, wave.

### Transitions into residential housing within 12 months of the final observation are crucial in predicting mortality

Among the objectives for this research was to examine whether or not differences exist in the mortality risk for BHPS members depending on their housing pathway. Model 3 in table 2 shows that after controlling for demographic characteristics, persons who moved to residential housing from their private homes 1 year before the end line measurement show the highest odds of mortality (OR 4.59, 95% CI 2.62 to 8.04). The group that transitioned to residential housing 1–2 years before the end line measurement also showed higher odds of mortality (OR 3.39, 95% CI 1.61 to 7.13) compared with those remaining in private housing.

Model 4 controls for health status at baseline. The odds of dying are attenuated across all housing transition categories. However, the overall pattern remains the same with persons who transitioned to residential housing 1 year before end line measurement being the most likely to die (OR 3.83, 95% CI 2.18 to 6.71). Transitions to residential housing 1–2 years before the end line measurement (OR 2.35, 95% CI 1.11 to 5.00) show higher odds than for sheltered transitions. The inclusion of socioeconomic measures (Models 5 and 6) makes modest changes to the mortality odds; the highest mortality risk is still for those transitioning to residential housing. Across the models, the BHPS wave is not statistically significant (and therefore not shown), yet it is necessary to include this variable to control for change in the risk of mortality through time across the sample. Housing tenure was tested but was not statistically significant (p=0.09). Finally, male mortality is higher in all models.

### Health and marital status have a key role in accounting for subsequent housing transitions and mortality in later life

Health status is important in accounting for the elevated mortality odds of persons who transition to residential housing. Those persons reporting poor or very poor health at baseline have very high odds (OR 3.89, 95% CI 2.97 to 5.09) of subsequent mortality, compared with those who report excellent, good or very good health. Marital status is an important predictor of mortality throughout the models, with persons who are single (never married) showing the highest odds of mortality (1.4 odds compared with those married and living as a couple). This is consistent with the literature on the intersection of marital status and mortality. Socioeconomic measures show those living in a household without a car have 1.2 greater odds of death. Transitions to widowhood (T0–T2) were tested but not statistically significant. Other household-related variables including the number of people in the household and a variable on living arrangements were tested but were not statistically significant. Although the effects of housing transitions seen in Model 3 are moderated by the inclusion of health and other variables, the evaluated risk of mortality in the 12 months following a move to residential housing remains significant.

### Male mortality is higher across all housing transition types

**Figure 2:**
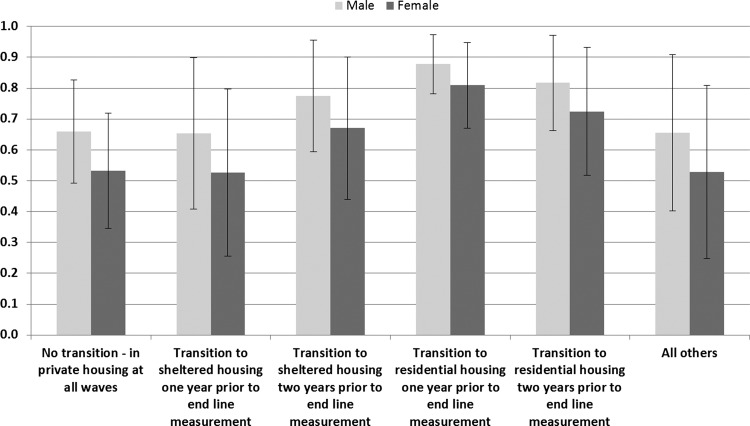
Predicted probabilities (and 95% CIs) of death between T2 and T3 for men and women by type of housing transition. T0=1994, aged 90+ years, never married, poor or very poor health status, manual and unskilled occupational class and missing/not answered for access to a car.

Predicted probabilities of death were calculated for men and women. These show higher male mortality across all housing transitions. The illustrated example in figure 2 combines the strongest predictors of mortality to show that persons moving into residential housing have the highest risk of mortality. However, among those who moved to residential housing within 1 year of end line measurement, the mortality differentials between men and women are narrower than among other trajectory types. Transitions into sheltered housing are not statistically significant compared with no transition (p>0.10). This reflects the different role of sheltered housing within the suite of housing options for older people—sheltered housing provides a longer-term form of housing which may account for similarities between remaining in private housing (no transition) and the less than 1 year sheltered housing group which is composed of persons more recently transferring from private housing.

## Discussion

Understanding the relationship between housing transitions and older people's mortality risk is important in a policy context which provides alternatives between different types of long-term care accommodation. The study has used a key explanatory variable that combines housing moves from the private sector into residential and sheltered housing, with the duration of stay in order to examine mortality. The highest risk of mortality was associated with transitions to residential housing in the previous 12 months. The mortality risks of those who moved to residential housing 12–24 months prior to the final observation (and therefore by definition had survived the first 12 months in residential housing) were somewhat lower, although still significantly above those who had remained in the private housing sector throughout. Transitions into sheltered housing were not statistically significant in the final model controlling for the full range of characteristics.

It is likely that as identified previously,^30^ there is a clear preference among older people to remain in their private housing until their care needs render a move into long-term care unavoidable. This may include transitions between private households which facilitate the provision of spousal or child-adult parent caring. Moves from private housing to residential housing may arise from the loss of household caring arrangements or deterioration of health among the older person.^11^ Using longitudinal data, Breeze *et al*, (1999)^9^ found that those in rented accommodation and in households without a car carried a higher mortality risk over 21 years of follow-up and also a higher risk of being in an institution. For men, being single was a major predictor of moving to an institution, and losing household access to a car was a strong factor for mortality or institutionalisation. In the present analysis housing tenure did not provide statistically significant results and was not included in the final analyses.

Our findings support previous research on the interaction of marital status and health that have consistently shown that unmarried persons experience adverse health and mortality outcomes. Unmarried persons experience poorer health and mortality outcomes than their married counterparts, which is consistent with existing literature.^16^ The results also highlight the importance of health status in accounting for transitions into residential housing and the need to control for this to account for the mortality of persons transitioning to residential housing. A persistent gender gap disadvantaging men was identified across all housing pathways with the differential being greatest among those persons who stayed in private housing and who spent less than 12 months in sheltered housing prior to death. This relates to past research findings identifying a downward trend over time in one's chances of living with relatives rather than alone or in a couple, with institutional residents experiencing a higher mortality risk than those living alone, in a couple or living with relatives.^2^ This gender gap may arise from marital caring and spousal care roles that could relate to transitions observed.

One potential limitation of the study is that the analysis focused exclusively on intersector moves and did not consider within-housing sector transitions (eg, from one private household to another private household). It also excluded measures of housing quality and the exact timing of housing moves. A second limitation is the difficulty in measuring change in health in relation to the housing move and mortality. Accurately capturing health status and change in health around the time of housing moves is difficult. Our research conducted sensitivity analysis on the use of different measures; no substantial changes from baseline measurement were observed. Notwithstanding these limitations, the present study has sought to shed light on the relationship between mortality risk and earlier housing moves, and this information is not readily available in other datasets. Our results are consistent with previous research and confirm that, relative to sheltered housing, residential housing stays will tend to be shorter in duration, with elevated mortality risk especially in the first 12 months.

**What is already known on this subject?**

- Previous studies have focused on transitions into residential housing and mortality over a longer period or have focused on palliative care at the end of life.
- Higher mortality among residents of long-term care institutions compared with those living alone, in a couple or with relatives has been identified^2^ but has not taken into account the duration or residence, or compared residential and sheltered housing.

**What this study adds?**

- The present study uses an explanatory variable that combines housing (sheltered and residential housing) trajectories with the duration of stay in order to examine mortality risk in later life.
- The study uses data on annual transitions which is more realistic for the study of housing moves in later life compared with past research that has considered 10 year or month-on-month transitions.
- Transitions into sheltered and residential housing types have been compared side by side.
- Results show that the mortality risk is highest for those who have moved into residential housing during the previous 12 months. Health and marital status appear to have a stronger effect on mortality risk than an individual's socioeconomic position.
- Men experience higher odds of mortality than women across all housing trajectories.
- The results contribute to our understanding of the relationship between ill-health, socioeconomic status, living arrangements and housing transitions and the probability of dying.
- The study findings have implications for the estimation of older people's mortality risk according to the time they spend in different forms of long-term care accommodation, and can therefore inform the design of social policy in the area of long-term care.
